# Frequency and factors associated with flare-ups in lupus patients with lupus nephritis in French Guiana

**DOI:** 10.3389/fneph.2026.1972371

**Published:** 2026-10-12

**Authors:** Arriel Makembi Bunkete, Mamadou Mouctar Diallo, Jonathan Muamba Mutombo, Malika Belgrine, Gloria Ningaro Timem, Comlan Ney-nicaise Vigan, Mohamed Sidibe, Timothé Davodoun, Irénée Djiconkpode, Tanguy Gbaguidi, Franklin Samou Fantcho

**Affiliations:** 1 Department of Nephrology and Dialysis, University Hospital of French Guiana, Saint-Laurent-du-Maroni Site, French Guiana, France; 2 Department of Nephrology and Dialysis, University Clinics of Kinshasa, University of Kinshasa, Kinshasa, Democratic Republic of Congo; 3 Department of Nephrology, Dialysis and Kidney Transplantation, University Hospital of Guadeloupe, Guadeloupe, France; 4 Department of Nephrology and Dialysis, University Hospital of French Guiana, Cayenne, French Guiana, France; 5 Department of Internal Medicine, University Hospital of French Guiana (CHU de Guyane), Saint-Laurent-du-Maroni, French Guiana, France

**Keywords:** anti-dsDNA antibody, French Guiana, lupus flares, lupus nephritis, systemic lupus erythematosus

## Abstract

**Background:**

French Guiana ranks among the French overseas territories with the highest prevalence of systemic lupus erythematosus (SLE). However, local data on lupus flares and renal involvement remain limited. This study aimed to determine the frequency of lupus flares and identify associated factors among patients with lupus nephritis (LN) compared with those without renal involvement.

**Methods:**

We conducted an observational historical cohort study using data from the West Guyanese lupus cohort between January 2018 and December 2022. Patients with confirmed SLE were consecutively included and classified according to the presence or absence of LN. Clinical, biological, and immunological data were extracted from medical records. Lupus flare was defined as documented disease worsening requiring therapeutic intensification. Factors associated with flares were assessed using multivariable logistic regression.

**Results:**

Sixty-two patients were included, of whom 45 (72.6%) had LN. Overall, 53% experienced at least one flare. Flares occurred more frequently in patients with LN than in those without LN (62.2% vs. 29.4%). Higher anti-dsDNA and anti-Smith antibody levels, higher SLEDAI scores, fever, pleural effusion, and cutaneous manifestations were significantly associated with flares. In multivariable analysis, LN (OR 3.64; 95% CI 1.00–13.23; p=0.049) and higher anti-dsDNA antibody levels (p=0.033) were independently associated with flares.

**Conclusion:**

Lupus flares were frequent and occurred more often in patients with LN. LN and higher anti-dsDNA antibody levels were independently associated with flares, supporting close monitoring of patients with renal involvement to identify disease activity and optimize management.

## 1 Introduction

Systemic lupus erythematosus (SLE) is a chronic, multifactorial autoimmune disease resulting from complex interactions between genetic, hormonal, environmental, and immunological factors, leading to loss of immune tolerance, autoantibody production, and inflammation that can affect multiple organs (1). It mainly affects women of reproductive age (1). Its prevalence in France is 47 per 100,000 inhabitants, with a heterogeneous distribution across the country (2). French Guiana ranks third in terms of SLE prevalence among French regions, with one of the highest prevalence rates observed in the French overseas territories (86.2/100,000) (3). Systemic lupus has a spectrum of organ involvement and severity, ranging from minimal skin involvement to severe cerebral, cardiac, or renal damage (4). However, data on the profile of organ involvement are insufficient in certain high-prevalence territories such as French Guiana. The disease course is characterized by episodes of remission and relapse (5). Lupus flares are episodes of disease worsening that may require treatment intensification. They may be multifactorial, with adverse consequences in terms of organ function prognosis and infectious complications, particularly in patients with kidney damage (6). The Guyanese context is unique: the population has cosmopolitan ancestral origins; almost half of the population lives below the poverty line, with frequent food insecurity; access to healthcare is often delayed for the most disadvantaged, particularly access to specialists; and the epidemiological transition from infectious diseases to chronic noncommunicable diseases is recent, but digestive parasitosis remains prevalent, particularly in the West. In this context, the incidence and presentation of the pathologies observed in French Guiana are often singular. Therefore, this study aimed to determine the frequency of systemic lupus flares and investigate associated factors, particularly lupus nephritis.

## 2 Methods

### 2.1 Study design and setting

This was an observational historical cohort study conducted at the University Hospital of French Guiana, Saint-Laurent-du-Maroni site, from January 2018 to December 2022. The study was based on a historical cohort of patients with SLE routinely followed at this institution.

### 2.2 Participants

All adult patients (≥18 years) with a confirmed diagnosis of systemic lupus erythematosus (SLE) according to the 2019 EULAR/ACR classification criteria were eligible. A minimum of 6 months of documented follow-up was required for inclusion in the study. Patients with incomplete medical records, uncertain diagnosis, or insufficient follow-up data were excluded. Participants were classified into two groups:

1. LN group: Patients with renal involvement (proteinuria > 0.5 g/L and/or hematuria > 10/mm^3^ or red cell casts).
2. Non-LN group: Patients without renal involvement.

### 2.3 Data collection and variables

Clinical, demographic, and biological data were retrospectively extracted from patient medical records using a standardized collection sheet.

### 2.4 Variables collected included

- Sociodemographic: age, sex, BMI, smoking status.
- Clinical: fever, arthritis, cutaneous involvement (malar rash, discoid rash, vespertilio), serositis, oral ulcers, hypertension, infections, and disease activity (SLEDAI score).
- Biological: complement levels (C3, C4), anti-dsDNA, anti-Smith, ANA, Coombs test, renal function tests, and inflammatory markers.

The primary outcome was the presence of at least one documented lupus flare, defined as disease worsening requiring therapeutic intensification (immunosuppression or corticosteroid escalation).

Remission was defined according to the clinical status documented in the patients’ medical records by the treating physician.

### 2.5 Bias and data quality control

To minimize selection bias, all eligible patients from the registry during the study period were included. Information bias was limited by using standardized data extraction by two independent investigators and cross-verification of discordant entries. Confounding factors were addressed through multivariable logistic regression.

### 2.6 Sample size and power

All eligible patients with complete records were consecutively included during the study period. Due to the limited population of lupus patients in French Guiana, the study size reflected the entire accessible cohort rather than a pre-calculated sample.

### 2.7 Statistical analysis

Quantitative variables were expressed as means ± standard deviations or medians [Q25–Q75], as appropriate, and qualitative variables as frequencies and percentages. The normality of continuous variables was assessed using the Shapiro–Wilk test. Comparisons between groups were performed using the χ² test or Fisher’s exact test for categorical variables and Student’s t-test for normally distributed continuous variables or the Mann–Whitney U test for non-normally distributed continuous variables. A multivariable logistic regression model was constructed to determine factors independently associated with lupus flares. Variables with p < 0.20 in univariate analysis were included in the model. The final multivariable model included lupus nephritis, anti-dsDNA antibody levels, and anti-Smith antibody levels. The strength of association was expressed as odds ratios (ORs) with 95% confidence intervals (CIs). Missing data was handled by complete-case analysis. Analyses were performed using Stata version 16.0 (StataCorp, College Station, TX, USA).

## 3 Results

### 3.1 Patient general characteristics

**Figure 1** shows that 72.6% of patients had lupus nephritis, whereas 27.4% had no lupus nephritis.

**Figure 1:**
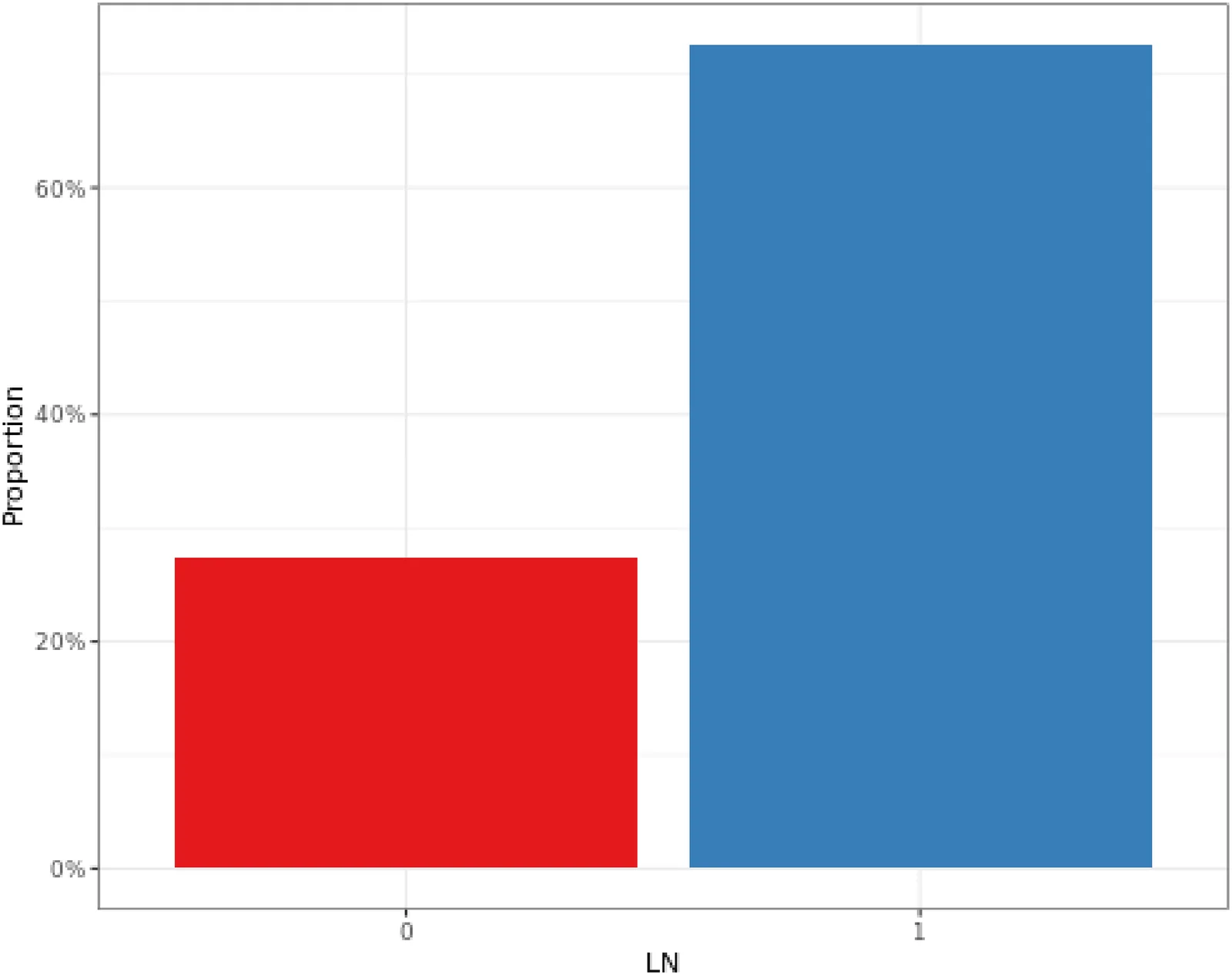
Population distribution according to lupus nephritis status.

**Table 1** shows that the two groups were comparable in terms of clinical features, except for joint involvement, discoid rash, and malar rash, which were significantly different between the two groups.

**Table 1:** Clinical characteristics of patients according to renal involvement.

Variable	Units	Without lupus nephritis (n=17)	With lupus nephritis (n=45)	n	P-value
Age	Median [Q25–Q75]	37.0 [24.0–47.0]	26.0 [20.0–37.0]	62	0.087
BMI	Mean (SD)	31.0 (13.1)	26.7 (15.8)	62	0.29
SLEDAI score	Median [Q25–Q75]	16.0 [14.0–19.0]	20.0 [14.0–24.0]	62	0.16
Alopecia	n (%)	5 (29%)	25 (56%)	30	0.066
Ascites	n (%)	1 (5.9%)	3 (6.7%)	4	1.00
Convulsions	n (%)	0 (0%)	5 (11%)	5	0.31
Joint involvement	n (%)	13 (76%)	43 (96%)	56	0.043
Pericardial effusion	n (%)	3 (18%)	10 (22%)	13	1.00
Pleural effusion	n (%)	4 (24%)	9 (20%)	13	0.74
Photosensitivity	n (%)	10 (59%)	28 (64%)	38	0.73
Fever	n (%)	7 (44%)	18 (40%)	25	0.79
Arterial hypertension	n (%)	7 (41%)	19 (42%)	26	0.94
Bacterial infection	n (%)	10 (59%)	27 (60%)	37	0.93
Viral infection	n (%)	1 (5.9%)	3 (6.7%)	4	1.00
Discoid rash	n (%)	8 (47%)	40 (89%)	48	<0.01
Female sex	n (%)	16 (94%)	40 (89%)	56	1.00
Smoking	n (%)	1 (5.9%)	5 (11%)	6	1.00
Oral ulcers	n (%)	2 (12%)	9 (20%)	11	0.71
Malar rash	n (%)	5 (29%)	31 (69%)	36	<0.01

BMI, body mass index; SLEDAI, Systemic Lupus Erythematosus Disease Activity Index; SD, standard deviation; Q25–Q75, 25th–75th percentiles.

**Table 2** shows that most biological parameters were comparable between patients with and without lupus nephritis. However, anti-dsDNA antibody positivity, ANA positivity, and haptoglobin levels differed significantly between the two groups (p=0.019, p<0.01, and p=0.044, respectively).

**Table 2:** Biological characteristics of patients according to renal involvement.

Variable	Units	Without lupus nephritis (n=17)	With lupus nephritis (n=45)	N	P-value
Albumin	g/L	30.9 [23.0–37.0]	27.0 [21.0–36.0]	62	0.24
Bilirubin	µmol/L	6.00 [5.00–11.0]	6.00 [4.00–8.00]	62	0.34
C3	U/mL	0.889 (0.411)	0.827 (0.392)	62	0.60
C4	U/mL	0.090 [0.070–0.190]	0.140 [0.080–0.230]	62	0.28
CH50	U/mL	43.0 [34.5–50.0]	42.0 [26.0–52.0]	62	0.73
Creatine phosphokinase	U/L	109 [67.0–249]	80.0 [45.0–190]	62	0.24
Creatinine	µmol/L	62.0 [54.0–77.0]	63.0 [54.0–98.0]	62	0.67
C-reactive protein	mg/L	9.10 [0.80–37.9]	12.3 [2.40–29.5]	62	0.95
White blood cell count	/mm^3^	5240 [3560–6620]	4760 [3511–6820]	62	0.93
Haptoglobin	g/l	1.10 [0.98–1.30]	1.40 [1.03–2.01]	48	0.044
Hemoglobin	g/dl	11.0 [8.90–12.1]	10.3 [8.60–11.3]	62	0.42
Lactate dehydrogenase	IU/L	260 [225–348]	284 [230–357]	62	0.60
Platelets	/mm^3^	225588 (146938)	255422 (116879)	62	0.46
APTT	Ratio	1.15 [0.97–1.25]	1.07 [1.00–1.21]	62	0.66
Prothrombin time	%	100 [87.0–100]	100 [86.0–100]	62	0.74
Urea	mmol/L	2.70 [2.10–4.80]	4.80 [2.90–6.90]	62	0.061
Anti-dsDNA antibodies	n (%)	8 (47%)	35 (78%)	43	0.019
ANA	n (%)	11 (65%)	43 (96%)	54	<0.01
Rheumatoid factor	n (%)	0 (0%)	2 (4.4%)	2	1.00
Anti-Smith antibodies	n (%)	8 (47%)	27 (60%)	35	0.36
Anti-SSA antibodies	n (%)	6 (35%)	25 (56%)	31	0.15
Anti-SSB antibodies	n (%)	2 (12%)	8 (18%)	10	0.71
Antiphospholipid antibodies	n (%)	1 (5.9%)	9 (20%)	10	0.26
Positive Coombs test	n (%)	1 (5.9%)	13 (29%)	14	0.087
Blood group O+	n (%)	9 (53%)	29 (64%)	38	0.46
Blood group B+	n (%)	4 (24%)	7 (16%)	11	–
Blood group A+	n (%)	2 (12%)	7 (16%)	9	–
Blood group O−	n (%)	2 (12%)	1 (2.2%)	3	–
Blood group B−	n (%)	0 (0%)	1 (2.2%)	1	–
Anti-U1RNP antibodies	n (%)	8 (47%)	27 (60%)	35	0.36

ANA, antinuclear antibodies; APTT, activated partial thromboplastin time; CH50, total hemolytic complement; C3, complement component 3; C4, complement component 4; dsDNA, double-stranded DNA; IU, international units; U1RNP, U1 ribonucleoprotein.

**Table 3** shows the comparison of patient outcomes according to the occurrence of lupus flare-ups. While mortality and dialysis rates did not differ significantly between groups, infectious complications and progression to chronic kidney disease (CKD) were markedly more frequent among patients who experienced flares (*p* < 0.001 and *p* < 0.01, respectively). The rate of clinical remission was slightly higher in patients without flares, although the difference was not statistically significant. These findings indicate that infection and renal deterioration were more frequent among patients who experienced lupus flares.

**Table 3:** Patient parameters following lupus flare-up.

Variable	Units	NoFlare(n=29)	Flare(n=33)	Pvalue
Death	n(%)	3(10%)	26(79%)	0.097
Dialysis	n(%)	2(6.9%)	8(24%)	0.088
Infection	n(%)	10(34%)	27(82%)	<0.001
CKD	n(%)	5(17%)	19(58%)	<0.01
Remission	n(%)	20(69%)	17(52%)	0.16

CKD, chronic kidney disease.

The figure shows that lupus flares were present in 53% of the study population.

**Table 4** summarizes the comparison of clinical and biological parameters between patients with and without lupus flares. Patients who experienced flares had significantly higher median anti-Smith and anti-dsDNA antibody levels and SLEDAI scores, as well as higher frequencies of pleural effusion, fever, discoid rash, oral ulcers, and malar rash.

**Table 4:** Clinical and biological parameters according to the occurrence of lupus flares.

Variable	Summary measure	No flare (n=29)	Flare (n=33)	P-value
Anti-Smith antibody level (UR/mL)	Median [Q25–Q75]	4 [2–73]	19 [5–137]	**0.048**
Anti-dsDNA antibody level (IU/mL)	Median [Q25–Q75]	16 [9–56]	47 [12–228]	**0.028**
SLEDAI score	Median [Q25–Q75]	15 [12–20]	22 [16–25]	**0.004**
Pleural effusion	n (%)	1 (3.4%)	12 (36%)	**<0.01**
Fever	n (%)	5 (18%)	20 (61%)	**<0.001**
Discoid rash	n (%)	18 (62%)	30 (91%)	**<0.01**
Oral ulcers	n (%)	1 (3.4%)	10 (30%)	**<0.01**
Malar rash	n (%)	12 (41%)	24 (73%)	**0.013**

dsDNA, double-stranded DNA; Q25–Q75, 25th–75th percentiles; SLEDAI, Systemic Lupus Erythematosus Disease Activity Index; bold values indicate statistically significant differences between the groups (p < 0.05).

The figure shows that Lupus flares were more frequent in patients with kidney damage (62.22%) than in those without kidney damage (29.41%).

In multivariable logistic regression analysis including lupus nephritis, anti-dsDNA antibody levels, and anti-Smith antibody levels, lupus nephritis was independently associated with the occurrence of lupus flares (OR 3.64, 95% CI 1.00–13.23; p=0.049). Higher anti-dsDNA antibody levels were also independently associated with flares (OR 1.01, 95% CI 1.00–1.01; p=0.033), whereas anti-Smith antibody levels were not significantly associated with flares (p=0.369) (**Table 5**).

**Table 5:** Multivariable logistic regression analysis of factors associated with the occurrence of lupus flares.

Variable	OR	SE	Z	P	CI95
Lupus nephritis	3.64	2.40	1.96	0.049	1.00–13.23
Anti-dsDNA antibody level	1.01	0.00	2.13	0.033	1.00–1.01
Anti-Smith antibody level	1.00	0.00	0.90	0.369	0.99–1.00
Constant	0.23	0.15	-2.33	0.020	0.07–0.79

OR, Odds Ratio; SE, Standard Error; P, p-value; CI95, 95% Confidence Interval; LN, Lupus Nephritis; AntiDNA, anti–double-stranded DNA antibodies; AntiSM, anti-Smith antibodies.

## 4 Discussion

The present study aimed to determine the frequency and identify factors associated with systemic lupus flares in patients with lupus nephritis in a Guyanese cohort, including 62 patients, of whom 72.58% had lupus nephritis and 27.42% did not (**Figure 1**). The two groups were comparable in terms of their clinical, biological, and therapeutic characteristics (**Tables 1**, **2**). Fifty-three percent of the population experienced at least one lupus flare. Lupus flares occurred in 62.22% of patients in the lupus nephritis subgroup versus 29.41% in the subgroup without lupus nephritis. The following clinical and laboratory parameters were significantly different between patients with and without lupus nephritis: anti-dsDNA and antinuclear antibody positivity, haptoglobin levels, joint involvement, discoid rash, and malar rash. Higher levels of anti-dsDNA and anti-Smith antibodies, fever, pleural effusion, discoid rash, malar rash, oral ulceration, and higher SLEDAI scores were significantly associated with the occurrence of flares. According to the multivariable logistic regression analysis adjusted for anti-dsDNA and anti-Smith antibody levels, lupus nephritis and anti-dsDNA antibody levels were significantly associated with systemic flares. Regarding clinical outcomes, infection and progression to chronic kidney disease were significantly more frequent among patients who experienced flares.

The frequency of lupus nephritis in our study was 72.58%, which was higher than that reported in the literature. Indeed, in an international cohort study published in February 2016 that included 1,827 patients, the frequency was 38.3%, among whom 80.9% had a diagnosis at inclusion, with an average follow-up time of six months before inclusion (7). This difference can be explained by the size of our sample, the monocentric and historical nature of the cohort, and the difficult access to care in French Guiana, which often leads patients to consult late. However, a more recent study published in 2020 in the U.S. suggested a slightly greater and more heterogeneous incidence depending on the population, compared with the international cohort (8). These findings underscore geographical disparities in the frequency and complications of lupus nephritis.

Of the total population, 53% had at least one history of lupus flare (**Figure 2**). The course of lupus is characterized by episodes of remission and relapse (**Table 3**). According to an Indian study published in 2015, 71.69% of patients experienced at least one relapse over a 6-month period (11). Despite current therapeutic advances, relapse remains inherent to the natural history of lupus and can be of variable severity (10, 13–16). In our cohort, lupus flares were more frequent in patients with renal involvement than in those without renal involvement (62.22% vs. 29.41%) (**Figure 3**). These results corroborate other findings reported in the literature, notably in the 20-year Spanish cohort, in which the incidence of flares was 38%, 58.6% of which were present in patients with lupus nephritis at the time of diagnosis (9). Another study also revealed that 30–40% of relapses involve at least two organs, with the kidney being the most frequently involved (10).

**Figure 2:**
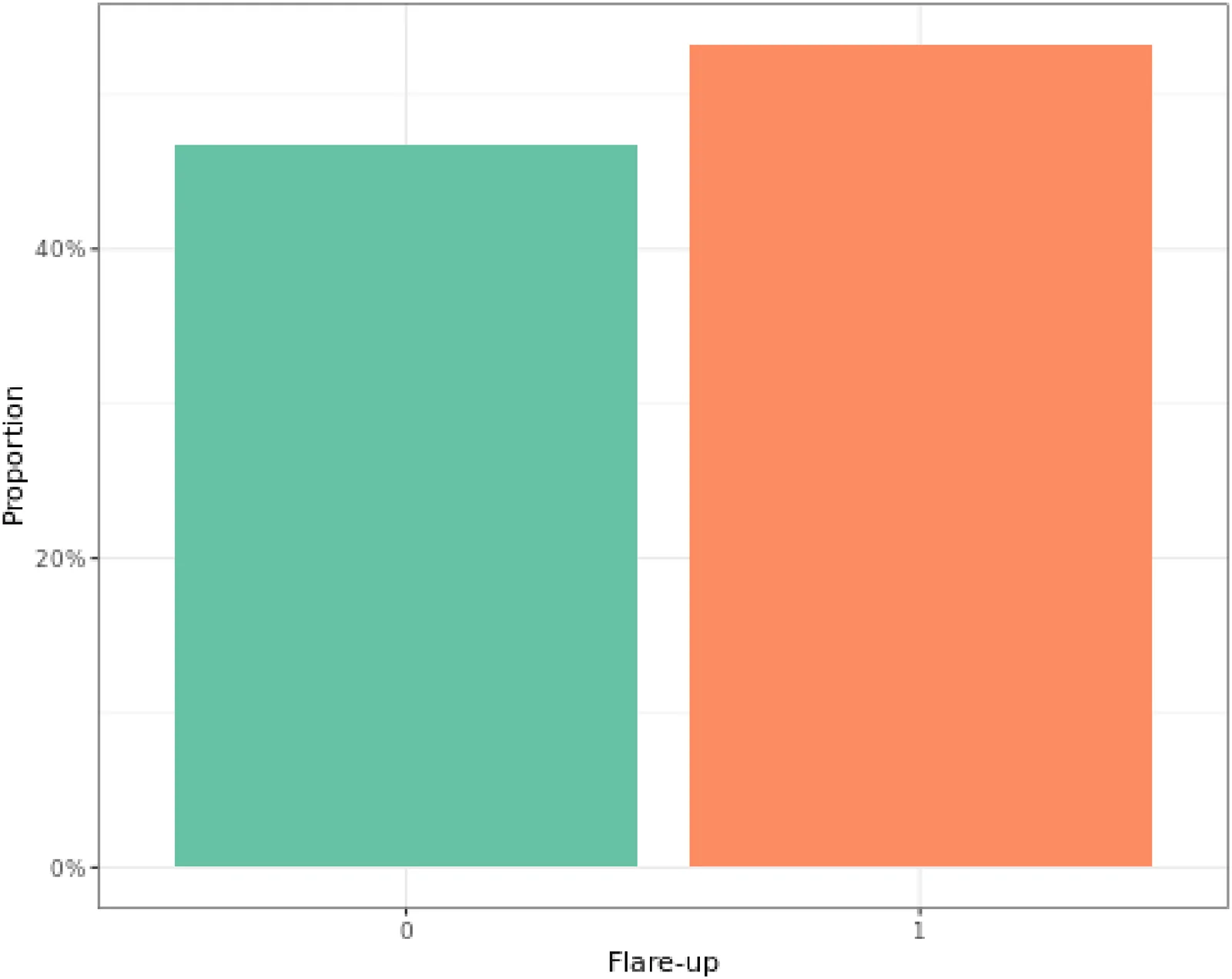
Distribution of lupus flare-ups.

**Figure 3:**
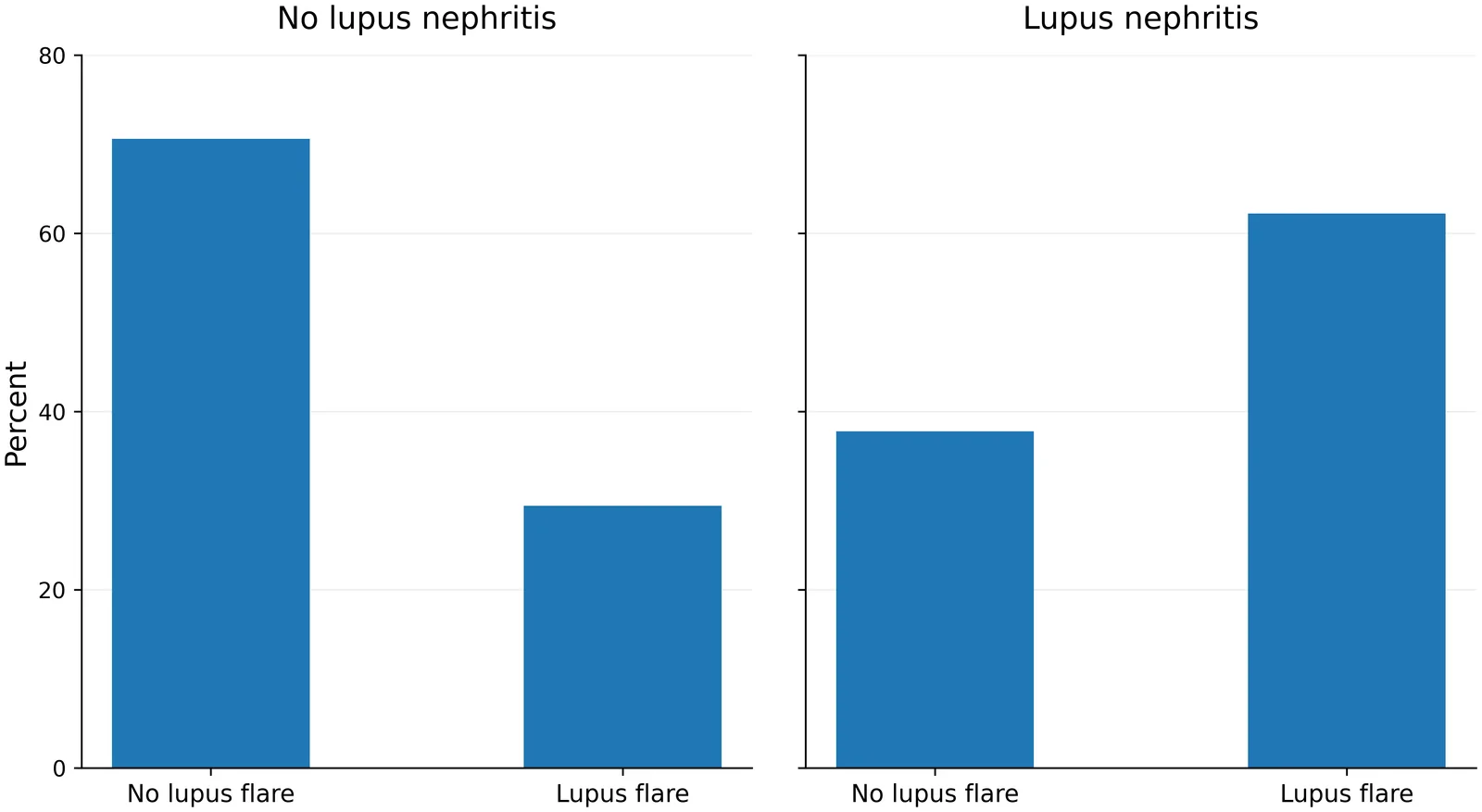
Distribution of lupus flares according to renal involvement.

The clinical and laboratory parameters that were significantly different between patients with and without lupus nephritis included anti-dsDNA and antinuclear antibody positivity, haptoglobin levels, joint involvement, discoid rash, and malar rash (**Tables 1**, **2**). These findings are consistent with other observations described in the literature, in which anti-dsDNA antibody positivity has been associated with renal involvement and overall disease activity (14, 17, 18).

The factors significantly associated with lupus flares in the present study were higher anti-dsDNA and anti-Smith antibody levels, fever, pleural effusion, discoid rash, malar rash, oral ulceration, and higher SLEDAI scores. These factors have also been associated with disease activity or flares in other studies worldwide (12). A South Korean study published in 2023, which assessed the risk of relapse in relation to antibody positivity, revealed that double positivity for anti-dsDNA and anti-Smith antibodies at diagnosis was associated with an increased risk of relapse (14).

Our logistic regression model investigating the association between lupus nephritis and lupus flares and adjusted for anti-dsDNA and anti-Smith antibody levels showed that lupus nephritis was independently associated with the occurrence of flares. Higher anti-dsDNA antibody levels were also independently associated with flares (**Table 5**). Indeed, as discussed above, the frequency of flares is higher in patients with lupus nephritis, depending on the initial degree of organ damage (8, 10). This finding is in line with previous studies highlighting the relationship between renal involvement and lupus disease activity. Although several studies have demonstrated that disease activity and relapse episodes persist in patients with end-stage kidney disease receiving dialysis, according to certain risk factors (20), several other studies have reported clinical and hematological remission of lupus, or at least a significant reduction in disease activity and relapse in patients with end-stage kidney disease receiving dialysis, even after therapeutic minimization (19, 20).

Finally, regarding clinical outcomes, the study noted a significantly greater frequency of infection and progression to chronic kidney disease in patients who had experienced flares. Indeed, it has been widely demonstrated that a high frequency of flares is associated with organ damage and progression to end-stage renal disease in patients with lupus nephritis (21). Moreover, the high frequency of infection may be related to immunosuppression resulting from the intensification of immunosuppressive therapy during flare episodes (22). Mortality was also particularly high among patients who experienced flares (79% versus 10% among patients without flares). Although this finding should be interpreted cautiously given the small sample size and retrospective design, it may reflect the greater disease severity among patients experiencing flares, together with the high burden of infectious complications and renal deterioration observed in this group. Delayed access to specialized care in French Guiana may also contribute to more severe clinical presentations. However, the observational design of the present study does not allow a causal relationship between lupus flares and mortality to be established. Given the high frequency of renal involvement observed in our cohort, these findings provide valuable data from French Guiana, a geographic setting in which data on lupus nephritis remain limited, and complement current evidence emphasizing the importance of renal involvement in SLE (23).

### 4.1 Study strengths and weaknesses

Like all retrospective studies, our study was affected by missing data, which, combined with the monocentric nature of the study, reduced the amount of available data for some analyses. In many patients, SLE was first diagnosed during hospitalization following the presentation of clinical manifestations suggesting the disease. Because the actual onset of SLE before hospitalization could not be reliably determined from the medical records, disease duration at hospitalization and the interval from disease onset to diagnosis could not be accurately assessed. As the diagnosis of lupus flare was based on documentation in the medical records, it was not possible to retrospectively compare all documented flares with contemporary standardized criteria for defining lupus flares. Despite these limitations, our study is the first to assess the association between initial renal involvement and systemic lupus flares in French Guiana, a territory with a high prevalence of lupus. In addition, remission was based on the clinical status documented in the medical records rather than on the DORIS definition. Owing to the retrospective nature of the study and the available data, LLDAS criteria could not be reliably assessed. This limits direct comparison with studies using contemporary treat-to-target definitions of remission and low disease activity.

## 5 Conclusions

Lupus flares were frequent in this cohort and occurred more frequently in patients with lupus nephritis than in those without renal involvement. In multivariable analysis, lupus nephritis and higher anti-dsDNA antibody levels were independently associated with the occurrence of flares. Patients experiencing flares also had higher frequencies of infection and progression to chronic kidney disease. These findings highlight the importance of close monitoring of patients with lupus nephritis, particularly those with elevated anti-dsDNA antibody levels, to identify disease activity and optimize management.

## Data Availability

The original contributions presented in the study are included in the article/supplementary material. Further inquiries can be directed to the corresponding author.
